# The Association between Fear of COVID-19 and Health-Related Quality of Life: A Cross-Sectional Study in the Greek General Population

**DOI:** 10.3390/jpm12111891

**Published:** 2022-11-11

**Authors:** Nikolaos Kontodimopoulos, Effimia Poulaki, John Fanourgiakis, Michael A. Talias

**Affiliations:** 1 Faculty of Social Sciences, Hellenic Open University, 26335 Patras, Greece; 2 Healthcare Management Program, School of Economics & Management, Open University of Cyprus, 2220 Nicosia, Cyprus; 3 Department of Management Science and Technology, Hellenic Mediterranean University, 72100 Agios Nikolaos, Greece

**Keywords:** FCV-19s, health-related quality of life, pandemic, COVID-19, SF-12, physical health, mental health, Greece

## Abstract

The aim of this cross-sectional study was to assess the level of fear related to the SARS-CoV-2 virus and the association of fear, and of sociodemographic and clinical characteristics, with health-related quality of life (HRQoL). A large sample of the Greek general population (N = 583) completed the validated versions of the Fear of COVID-19 scale (FCV-19s) and the 12-item Short Form (SF-12), and provided data on socio-demographic status, health history and COVID-19 protective behaviors. Variables were compared with Mann-Whitney and Kruskal-Wallis tests and associations with Spearman’s correlations. Gamma regression models investigated the influence of sociodemographic and COVID-related variables on HRQoL. The mean FCV-19s score for the sample was 18.3 ± 5.6, and physical and mental component summary scores were 50.2 ± 7.9 and 46.7 ± 10.1, respectively. More fear of COVID-19 was expressed by females (*p* < 0.001), individuals with comorbidities (*p* < 0.01), those with contacts with comorbidities (*p* < 0.001), and individuals not having caught COVID-19 (*p* < 0.05). Contrastingly, less fear was expressed by unvaccinated individuals and those with less frequent intake of information about the pandemic. Item level and overall FCV-19s scores were negatively associated with SF-12 summary scores, and fear of COVID-19 was the most important predictor of both physical and mental HRQoL. The findings from this and other similar studies could help to identify specific population groups in need of interventions to improve their physical and mental health, which had deteriorated due to the pandemic.

## 1. Introduction

Fear, anxiety, and stress are normal responses to perceived or real threats, particularly at times when people are faced with uncertainty or the unknown. Thus, it is normal and understandable that people are experiencing fear in the context of the COVID-19 pandemic. Furthermore, it is known that excessive and prolonged fear could lead to functionality reduction and impaired mental health [1]. In addition to the fear of contracting the virus in a pandemic, there are significant changes implemented in daily life, as movements are restricted to support efforts to contain and slow the spread of the virus. In most countries, new realities such as working from home, temporary unemployment, the home-schooling of children, and a lack of physical contact with family members, friends and colleagues, highlight the importance of looking after the mental and physical health of the population. Apart from the effect on health, outbreaks of infectious diseases negatively affect social functioning of individuals and societies, and have significant economic consequences as well [2,3].

Experience from past outbreaks (e.g., SARS and Ebola) shows that the associated fear can have psychological consequences such as social exclusion of positively tested patients, survivors, their families and other individuals associated with the disease, and provoke mental health problems [4,5]. Recent research has focused on identifying the potential implications of COVID-19 on physical and mental health in different segments of the population [6,7]. It is therefore important to study the relationship between the fear associated with the pandemic and health-related quality of life (HRQoL). In addition to problems directly caused by the virus, psychosomatic symptoms involving fear and anxiety about being infected and infecting others are potentially worrying, which justifies studies on the incidence of mental health symptoms, in the general population, and in subgroups of the population [8].

The aim of this study was to assess the level of fear related to the SARS-CoV-2 virus and to assess the association of this fear, and of sociodemographic and clinical characteristics, with HRQoL in a large sample of the Greek general population. As the long-term effects of COVID-19 are not yet known, this study could contribute to formulating policies and interventions to alleviate the growing health crisis in a post COVID-19 society. By gaining a deeper understanding of how this pandemic has affected individuals, the findings could help to identify what kind of support is needed to improve quality of life in these areas. Knowing the associated level of fear in different population groups will help to understand whether education and prevention programs are needed, and by which target groups, in order to increase overall quality of life [9].

## 2. Materials and Methods

### 2.1. Sample and Data Collection

The sampling goal of this cross-sectional study was to find a large sample of willing adult participants from the Greek general population who can read and understand Greek and to collect data in a relatively fast and affordable way. The urban city of Veria, which is located in the geographic region of Macedonia in northern Greece, and has a population of approximately 50,000, was the designated sampling unit. In the absence of a sampling frame, i.e., a complete and up-to-date list of the members of the population under investigation, two non-probability (convenience) sampling techniques were used: (i) voluntary sampling, where people volunteered themselves by responding to the survey questionnaire, which was distributed at public gathering points, e.g., supermarkets, pharmacies, COVID-19 vaccination points, etc., and (ii) snowball sampling, where the researchers initially contacted people who met the study’s inclusion criteria and, after completing the survey, were asked to recommend others who also meet the study criteria. A mixed sampling procedure involving convenience and snowball sampling was also used in a recent Greek study on the psychosocial consequences of the COVID-19 pandemic [10].

The questionnaire was distributed between November 2021 and January 2022, and the two sampling methods combined resulted in 583 participants. People under the age of 18 were excluded from the survey. Background information questions were included to obtain data on gender, age, family status, educational level, employment, comorbidities, hospitalizations, and on various COVID-19 characteristics like having been diagnosed with the virus, have been in contact with individuals who had been diagnosed with COVID-19, vaccination status, the frequency of information intake about the pandemic, etc. Self-reported changes in health status over the past year were also recorded with the stand-alone health transition item from the widely used SF-36 Health Survey. Participants with minor missing information on the sociodemographic characteristics were included in the subsequent analyses as they provided complete values on the self-report measures.

### 2.2. Instruments

Fear was measured by the Fear of COVID-19 Scale (FCV-19s) which is a self-report measure developed to determine the fear level of individuals during the COVID-19 outbreak. It consists of seven items assessing fear toward COVID-19, i.e., most afraid, feeling uncomfortable, clammy hands, fear of dying, feeling nervous or anxious, sleep problems, and heart palpitations. The items are rated on a 5-point Likert-type scale (1 = strongly disagree to 5 = strongly agree), and none are reverse scored. Higher overall scores correspond to higher levels of fear. The original scale showed very good internal consistency (Cronbach’s alpha = 0.82) and factor analysis confirmed the one factor solution proposed in the developmental study [6]. The instrument has been translated into Greek and was validated in a sample of 3029 individuals. The internal consistency of the Greek version was also found to be very high (Cronbach’s alpha = 0.87) [11].

General HRQoL was assessed with the SF-12, which is composed of 12 items derived from the eight dimensions of the SF-36, i.e., physical functioning, role physical, bodily pain, general health, vitality, social functioning, role emotional, and mental health, and has been shown to be reliable and valid [12]. The answer options involve unidirectional Likert-type scales to evaluate severity or frequency, and all 12 items are used to calculate both the physical and mental component summary scores (PCS-12 and MCS-12 respectively) using a scoring algorithm empirically derived from the data of a US general population survey [13]. It has been recommended that the US-derived summary scores, which yield a mean of 50 and a SD of 10, be used in order to facilitate cross-cultural score comparisons [14]. The Greek version of the SF-12 has been tested in a representative sample of 1005 members of the Greek general population, and has demonstrated good construct validity [15].

### 2.3. Statistical Analyses

Demographic and health-related variables of the sample were summarized using descriptive statistics. The normality of the distributions was assessed with the Kolmogorov-Smirnov test, based on which comparisons between subgroups were made with nonparametric Mann-Whitney and Kruskal-Wallis tests. The Spearman coefficient was used to determine the level of correlation between the continuous variables. The SF-12’s physical and mental health scores are by definition non-negative, continuous, and positively skewed data and, due to this, multivariate analysis with Gamma regression was used to model the PCS and MCS scores. With each of the two SF-12 component scores as the dependent variable, the relationships between HRQoL, fear of COVID-19 and sociodemographic and health-related variables were investigated. All analyses were performed with IBM SPSS Statistics v.24.

### 2.4. Ethical Issues

The study was approved by the Institutional Review Board (RB) of the postgraduate program in Health Care Management of the Hellenic Open University (IRB approval reference number: 130800/19-9-2021). The research was carried out in accordance with the Declaration of Helsinki. All participants provided informed consent and were informed that they could withdraw from the study at any time.

## 3. Results

A total of 590 respondents participated in the survey, seven of which provided incomplete data and were excluded from the analyses, resulting in a final sample of 583 valid, completed questionnaires. The respondents’ sociodemographic and health-related characteristics are displayed in Table 1. The results show that the majority of the respondents were female (65.0%), married or living with a partner (68.6%), and employed (78.6%). The majority also had a high education level (59.0%). In terms of age, respondents between 45 and 54 years old were the largest group in the sample (31.5%), followed by the group between 35 and 44 years of age (23.4%) (mean  =  45.2; SD  =  13.6). Most participants (73.9%) reported that their health was roughly the same compared to a year ago. Regarding comorbid conditions, 15.1% reported at least one, which in 60% of the cases was diabetes.

Half of the sample (50.3%) reported that at least one family member had comorbidity, and a larger portion (84.7%) reported having family members and/or contacts currently suffering or having suffered from COVID-19. A fifth of the sample (21.2%) reported having contracted the virus, but only a small proportion (1.8%) required hospitalization. The majority (78.8%) was fully vaccinated against the SARS-CoV-2 virus. Most participants (77.0%) followed the developments (e.g., formal governmental announcements) regarding COVID-19 on a daily basis, or at least 2–3 times a week. The fear of COVID-19 score for the entire sample was 18.3, which is slightly higher than the corresponding value (16.2) from the Greek validation study [11]. As for HRQoL, PCS-12 and MCS-12 scores were 50.2 and 46.7 respectively, which are comparable to the values reported in the Greek SF-12 validation study (49.4 and 48.9 respectively) [15].

The clear association between fear of COVID-19 and HRQoL is confirmed by the data presented in Table 2. For all seven items of the FCV-19s, an increasing level of agreement with each statement (revealing increased fear of the coronavirus pandemic) is associated with a decreasing level of both physical and mental HRQoL, as shown by the SF-12 summary component scores (*p* < 0.001 for twelve out of fourteen comparisons performed with the Kruskal-Wallis test). It is also worth noting that for the “agree” and “strongly agree” response levels, all of the mean PCS-12 scores are below 50, which corresponds to the mean health of the general population, thus implying a deterioration of physical health for higher levels of fear. As for the MCS-12 scores, they were below the Greek population means even at the “strongly disagree” response level, implying a clear deterioration of mental health with increasing levels of fear of COVID-19.

Weak to moderate, but statistically significant (*p* < 0.001), inverse correlations were observed between all FCV-19s items and overall score, with the PCS-12 and MCS-12 summary scores (Table 3). Weak, positive correlations were recorded between age and fear of the coronavirus, i.e., increasing age was significantly (*p* < 0.05 or better) associated with higher levels of fear in four of the FCV-19s items, as well as in the overall score. The association between age and HRQoL was also significant, but the direction of the relationship was different for PCS-12 and MCS-12, as increasing age was negatively associated with physical health (rho = −0.289, *p* < 0.001), and positively with mental health (rho = 0.121, *p* < 0.01).

Fear of COVID-19 and physical and mental health were compared by demographic and health-related characteristics, and the results are presented in Table 4. Women demonstrated higher levels of fear of COVID-19 and worse physical and mental health than men (Mann-Whitney *p* < 0.001). The inverse relationship between age and physical health was confirmed by the decreasing levels of PCS-12 in adjacent groups of increasing age (Kruskal-Wallis *p* < 0.001). Family, education and work status affect fear of COVID-19 and physical (but not mental) health, as people who are single, with higher education, and who are employed expressed less fear and reported better physical health. Self-reported health deterioration over the past year was linked to increased fear of the coronavirus and worse physical and mental health (*p* < 0.001). People suffering from at least one comorbidity expressed greater fear (*p* < 0.01), poorer physical (*p* < 0.001) and mental (*p* < 0.05) health, but previous hospitalization for the comorbid condition only affected physical health (*p* < 0.001). Having family members, friends or contacts with a comorbidity resulted in increased fear and lower mental health (*p* < 0.001), but having contacts who had caught COVID-19 did not affect either fear or HRQoL.

**Figure 1:**
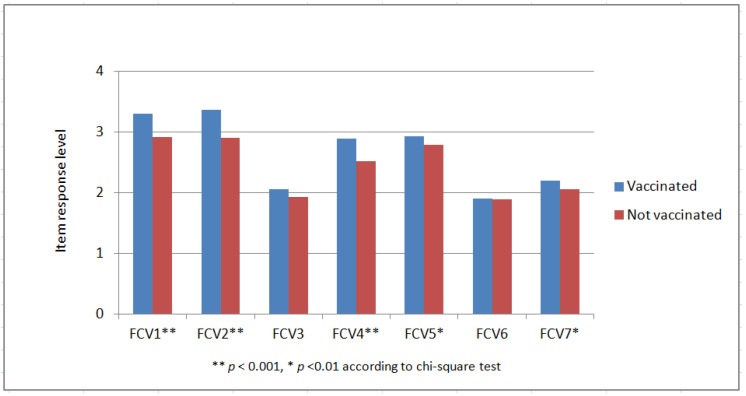
Distribution of FCV-19s responses by vaccination status.

**Figure 2:**
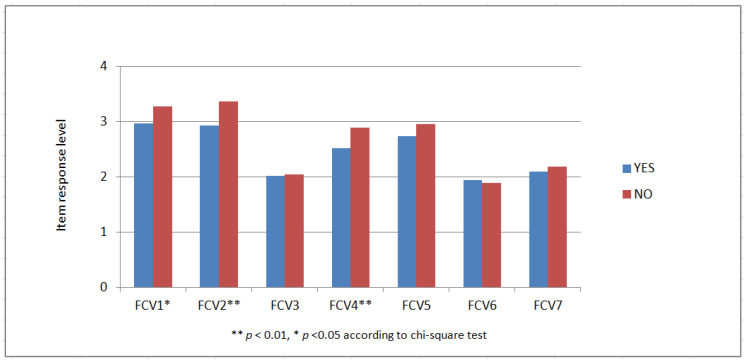
Distribution of FCV-19s responses by past Coronavirus infection (Yes/No).

The approximately 80% of the sample that was fully vaccinated expressed higher fear levels (*p* < 0.01). This is further elaborated in Figure 1, which shows that vaccinated individuals reported more fear than their unvaccinated counterparts on each of the seven fear dimensions measured by the FCV-19s instrument, and the differences in most cases were statistically significant (*p* < 0.01 or better). Furthermore, individuals who had previously caught the virus expressed more fear than those who had not (*p* < 0.05), and according to Figure 2, this is more evident in the FCV-19s items 1 (being afraid), 2 (being uncomfortable), 4 (being afraid of dying) and 5 (being anxious about COVID-19 news). On the other hand, having been hospitalized for COVID-19 did not appear to affect fear or physical/mental health. Finally, participants who were regularly updated about the virus and recent developments showed higher levels of fear and worse physical health (*p* < 0.001) than others who rarely or almost never received updates.

The results of the multivariate analysis with Gamma regression, which was used to model the PCS and MCS scores, are presented in Table 5. Higher fear of COVID-19, increasing age, having at least one comorbidity and previously hospitalized for this comorbid condition, female gender, being divorced or widowed and past year self-reported decreasing health were negatively associated with physical health. Fear of COVID-19 and age were the two strongest predictors of physical health. The results were quite similar in the MCS model, with the most noteworthy difference being the positive association between age and mental health.

The goodness of fit of the gamma regressions was assessed by examining the deviance residuals for both models. For the PCS-12 model, deviance residuals were between −0.494 and 0.344, with a median of 0.020, and first and third quartile values of −0.075, and 0.087, respectively. For the MCS-12 model, deviance residuals were between −0.922 and 0.569, with a median of 0.018, and first and third quartile values were −0.096 and 0.123, respectively. For both models, the QQ plots and residual diagnostics graphs were within acceptable limits (results not shown for parsimony).

## 4. Discussion

Millions of individuals all over the world have been infected by COVID-19, and many of them have eventually succumbed to COVID-19. Approximately three years after onset, the numbers of cases and deaths are continuing to rise in most countries, resulting in widespread fear and anxiety, and deteriorated psychological health and well-being. Increasing levels of anxiety since the beginning of quarantine have resulted in high levels of depression due to the impact of social isolation, growing infection rates, and increasing uncertainty [16]. Despite the eventual development of a vaccine which is easily available in most countries, the lack of an effective treatment method increases the feeling of fear even more, adding to existing health problems [17,18,19]. Health-related quality of life has also been adversely affected because of restrictions such as social isolation and distancing, wearing face coverings and not mixing households, in addition to the initially imposed long-term closure of the leisure, travel, retail, and hospitality sectors. Although these measures intended to decelerate the transmission of the virus, they have adversely affected the mental health and economic stability of many individuals [20].

In this study, fear of COVID-19 was the most important predictor of physical and mental HRQoL, a finding which is consistent with results from numerous studies reporting the association between these variables. For example, one study showed that people who experienced a history of fear, anxiety, depression, and stress reported lower physical and psychological health [21]. Also, fear of COVID-19 infection is associated with an increased feeling of insecurity, the fear of losing loved ones, increasing psychological distress, and decreasing HRQoL [22,23]. The COVID-19 fear score for our entire sample was 18.3 ± 5.6, which is very close to the respective score reported in a general population study in Turkey (18.13 ± 6.14) [24], higher (i.e., more fear) than scores from studies in France (15.82 ± 6.19) [25], Russia and eastern countries (17.2 ± 4.7) [26], Italy (16.86 ± 6.06) [27] and the Greek validation study (16.24 ± 5.04) [11], but lower (i.e., less fear) than the score from samples in Japan (21.24 ± 5.37) [28] and Bangladesh (21.38 ± 7.87) [29]. Differences in fear levels between countries may be due to variations in the sociodemographic characteristics of the samples, as well as the spread of the epidemic, government policies, and the level of information given to the public [30].

We found that fear of COVID-19 was higher among women, which is again consistent with most published findings [2,24,29]. A possible explanation for the gender difference might be that women are generally more susceptible to stress than men [31]. However, there are studies that have shown men to be more likely to experience depression than women [32], or that there is no gender difference in comparisons for stress and anxiety as a result of COVID-19 [33]. The older part of the sample (+65 years of age) had the highest fear scores among the age groups, which is consistent with the results from the Greek validation study [11]. In addition, fear of COVID-19 was found to be significantly associated with family status and educational level, as single people and those of higher education reported less fear on average. In our study, the more frequent intake of information regarding the COVID-19 pandemic was associated with higher levels of fear and lower HRQoL, which might be implying that the way in which the updates are presented over the media could be doing more harm than good, but also could be due to the aforementioned educational discrepancies.

The literature generally agrees that health literacy is lower among individuals with lower levels of education [34], and that it is difficult for these individuals to reach accurate information about the virus when their low health literacy is taken into account [35,36]. Hence, it might be expected that the level of fear is high in individuals who cannot obtain the correct information about COVID-19. Generally, health literacy has been shown to improve HRQoL during the COVID-19 epidemic [37]. In order to improve health literacy and control the corona virus and its consequences, governments need to provide the public with updated, timely, accurate, transparent, brief, simple information and knowledge regarding the pandemic [38].

Our results showed that people with a comorbid condition (mostly diabetes, but also coronary disease, hypertension, cancer, COPD, etc.), those with family/social contacts with a comorbid condition, and those who had been previously hospitalized for their comorbidity reported significantly higher levels of fear of COVID-19. These findings might have been expected, as these particular individuals are more prone to COVID-19 associated complications, leading to a drastic rise in morbidity and mortality. This has been confirmed in numerous studies involving patients with diabetes [39], cardiovascular disease [40], COPD [41], as well as other chronic illnesses. When a person with comorbidity is infected with SARS-CoV-2, the virus poses more of a threat, and managing these patients with adequate medical care is critical to their survival [42]. Co-morbid persons should adhere to preventive measures to reduce mortality, including regular hand-washing with soap or using an alcohol-based hand sanitizer, minimizing in person contact and practicing social distancing, wearing a face mask in public places, and avoiding public places [43].

In this study it was also shown that the 78.8% of the sample which had been vaccinated against the coronavirus expressed more fear than those who had not been vaccinated (regardless of the reason). This finding is consistent with most studies that have addressed this particular issue [44,45,46]. A typical characteristic of fear is that people tend to avoid what they fear, which might be implying that the negative association between the fear of COVID-19 and vaccine hesitancy is because people with high levels of fear of COVID-19 try to avoid what they fear most, i.e., in this case COVID-19, by getting vaccinated against it. However, in a study carried out in Lebanon [47], no significant association was found between the willingness to receive the COVID-19 vaccine and the fear of COVID-19, and the reasons for the difference might be cultural or due to a difference in sample size. On the other hand, our study did not reveal a significant difference in the level of fear between those who have a relative or friend in their immediate environment diagnosed with COVID-19 and those who do not, and a similar result was found in a recent study from Turkey [24].

Self-reported health transition over the past year was measured with the specific SF-36 Health Survey item, which asks respondents the amount of change in their health in general over a one-year period, and is based on the tested hypothesis that self-reported transitions reflect true changes in health during the recall period [48]. This item is not used to score any of the SF-36’s eight dimensions and is analyzed as a stand-alone ordinal variable. Evidence suggests that single-item measures capture a broad range of health dimensions, and reflect an enduring rating of one’s health status [49]. Our results showed that individuals who perceived their health status to be deteriorated expressed higher levels of fear of COVID-19. This agrees with the statistically significant negative correlations between physical and mental health, and all the FCV-19s items and with the overall fear score, which were observed in this study.

The multivariate analyses showed that the important predictors of physical and mental health in this study were quite similar. Higher fear of COVID-19, comorbidity and hospitalization due to comorbidity, female gender and self-reported decreasing health were negatively associated with both SF-12 summary scores. Fear was clearly the most important predictor for both outcome variables. Regarding physical health, some studies have suggested that the fear and anxiety associated with the COVID-19 outbreak causes physical symptoms which cannot be medically explained [50,51], or that those individuals with health problems avoid seeking health services for the fear of being infected with COVID-19 [52]. In addition, physical health is often impaired because people experienced difficulties in accessing nutrition and cleaning products due to lockdowns aimed at preventing the spread of the pandemic [24]. As for mental health, the fear of COVID-19 adversely affects psychological well-being and leads to anxiety, stress and depression [18,32,53], and the use of anxiolytic drugs has increased after the COVID-19 outbreak [54]. Interestingly, age was differently associated with physical and mental health, negatively with the former, and positively with the latter. Previous research suggests that older adults are more resilient than younger adults, and possessing higher emotional regulation and problem-solving approaches to cope with adversity [55].

Fear of COVID-19 affects the psychological health and well-being of health professionals as well, and the provision of appropriate education and training activities is of high importance to manage their stress levels. A Greek study investigated the effect of COVID-19 on the mental well-being of mental health workers and concluded that organizational support is required to protect their mental health and to ensure the quality of care they provide during prolonged crises, such as the current pandemic [56]. In a multicenter cross-sectional study conducted in five European countries (including Greece) before the start of mass vaccination, it was shown that nurses were more fearful of COVID-19 compared to nursing students [57]. Finally, another study examined the role of resilience and traumatic stress coping strategies for the healthcare workers following the first lockdown in Greece, and concluded that enhancing internal resources through supportive services will improve the workers’ ability to withstand, recover, and thrive with benefits to their psychological health and well-being [10].

Like in most countries, the COVID-19 pandemic has created fear in Greece as well. Our results, in agreement with most of the literature, show that pandemic-related fear adversely affects individuals’ physical and mental HRQoL, and strongly suggest the need for health and well-being interventions and programs to deal with the current and future effects of this crisis. Vaccination against COVID-19 was not a significant predictor of improved HRQoL in this study. Furthermore, protective measures (e.g., isolation, wearing masks and maintaining physical distance) have not produced the expected results in terms of reduced fear and better physical and mental health [58]. During and after the pandemic, attention should also be focused on non-pharmacological interventions such as stress and sleep control, healthy diet, physical activity, social connection, meaningful activity, and self-care skills, which are important and can significantly contribute to staying healthy. Policymakers could strengthen infrastructure to connect vulnerable populations to essential resources and services, deliver clear public health messaging, invest in better community infrastructure to encourage regular physical activity, and provide more programming to promote social cohesion. Public health, educational, and counseling programs might incorporate these strategies to support physical and mental health among vulnerable population groups [55].

This study has some limitations which should be considered. The sample, although relatively large, was recruited from one peripheral city in Greece, and the results cannot be generalized to the entire population. Based on the objectives of this research, which focuses on a sensitive topic, a multi sampling approach with two non-probability methods was used, i.e., voluntary and snowball sampling. Volunteer sampling is used in sensitive research when it is necessary to rely on those who are willing to answer requests to provide data. Snowball sampling expands sample size through referrals made by individuals who share a particular characteristic of research interest with the target population. Although both are relatively cheap, simple and cost-efficient ways to collect data, they give no true control over the makeup of the final sample as volunteers might share certain traits which differ from non-volunteers. Snowballing might extend the representativeness and bias problems because the initial participants tend to nominate people that they know well, regardless of their suitability to provide data. Finally, it should be noted that that a one-time cross-sectional study cannot capture the ongoing effects of the COVID-19 pandemic on various dimensions of HRQoL, and future research could focus on employing longitudinal designs to collect the data.

## 5. Conclusions

This study assessed the level of fear related to the SARS-CoV-2 virus and the association of this fear, and of sociodemographic and clinical characteristics, with HRQoL in a large sample of the Greek general population. Fear for COVID-19 was found to be higher in females, and positively correlated with age. Individuals with comorbidities and/or that are in contact with people with comorbidities, as well as those who had not caught COVID-19 in the past also expressed more fear. On the other hand, less fear was expressed by individuals who—for any reason—were not vaccinated against the virus and by those individuals with a less frequent intake of information on COVID-19. The FCV-19s scores, both overall and at item level, were negatively associated with the SF-12 physical and mental health summary scores, and fear for COVID-19 was the most important predictor of physical and mental health in the multivariate analyses. As the long-term effects of COVID-19 are still unknown, this and other similar studies could assist in policymaking, and in particular in formulating interventions to improve quality of life in the hopefully soon to come post-COVID era. Quantifying the level of fear in different population groups could be a positive step in the direction of identifying specific areas where education and prevention programs are required.

## Figures and Tables

**Table 1:** Characteristics of the sample (N = 583).

Characteristic	Mean ± SD	N (% Valid)	Characteristic	Mean ± SD	N (% Valid)
Gender female		379 (65.0)	Comorbidity (one or more)		88 (15.1)
Age (years)	45.2 ± 13.6		Hospitalized for comorbidity		8 (1.5)
Family status			Family/Contacts with comorbidity		289 (50.3)
Single		133 (22.8)	Family/Contacts with COVID-19		489 (84.7)
Married/Partner		400 (68.6)	Caught COVID-19		123 (21.2)
Divorced/Widowed		50 (8.5)	Hospitalized for COVID-19		10 (1.8)
Education			Vaccinated against COVID-19		457 (78.8)
≤9 years		58 (10.0)	Updated about COVID-19		
10–12 years		180 (31.0)	Regularly (e.g., daily)		303 (52.0)
≥12 years		342 (59.0)	Often (e.g., 2–3 times weekly)		146 (25.0)
Work status			Rarely (e.g., once weekly)		45 (7.7)
Employed		455 (78.6)	By chance (not pursued)		89 (15.3)
Housekeeping/Retired		76 (13.1)	FCV-19s	18.3 ± 5.6	
Unemployed		48 (8.3)	PCS-12 score	50.2 ± 7.9	
Health change (over the last year)			MCS-12 score	46.7 ± 10.1	
Much better/somewhat better		94 (16.4)			
Roughly the same		424 (73.9)			
Somewhat worse/much worse		56 (9.8)			

**Table 2:** Physical and mental component scores by FCV-19s response level.

FCV-19s Response Levels	FCV1		FCV2		FCV3		FCV4		FCV5		FCV6		FCV7	
	PCS	MCS	PCS	MCS	PCS	MCS	PCS	MCS	PCS	MCS	PCS	MCS	PCS	MCS
Strongly disagree	52.54	48.79	53.07	49.08	52.58	48.67	52.36	49.86	51.96	50.91	51.79	48.65	52.20	49.13
Disagree	52.05	48.87	51.47	49.93	50.01	47.15	52.86	48.61	51.44	49.74	49.83	46.37	51.03	47.60
Neither agree/disagree	50.73	47.60	50.71	48.19	48.80	45.06	50.26	46.23	50.32	47.47	49.22	45.00	48.65	45.09
Agree	49.37	46.15	49.96	45.01	47.02	41.89	47.21	44.90	49.76	43.55	46.16	42.05	47.15	42.83
Strongly agree	46.59	40.36	45.97	41.85	39.50	38.82	45.47	40.91	43.97	36.59	39.64	35.46	40.41	34.24
p-sig ^1^.	0.014	<0.001	0.002	<0.001	<0.001	<0.001	<0.001	<0.001	<0.001	<0.001	<0.001	<0.001	<0.001	<0.001

^1^ According to Kruskal-Wallis test, FCV1: most afraid, FCV2: uncomfortable, FCV3: clammy hands, FCV4: afraid of dying, FCV5: nervous/anxious, FCV6: can’t sleep, FCV7: heart palpitations.

**Table 3:** Spearman’s correlations between age, COVID-19 fear and SF-12 summary scores.

	FCV-19s	Age	FCV1	FCV2	FCV3	FCV4	FCV5	FCV6	FCV7
FCV-19s fear score	---	---	0.761 ***	717 ***	0.734 ***	0.805 ***	0.708 ***	0.703 ***	0.774 ***
Age	0.105 *	---	0.109 **	0.093 *	0.070	0.141 **	−0.012	0.112 **	0.054
Physical health (PCS12)	−0.277 ***	−0.289 ***	−0.148 ***	−0.149 ***	−0.240 ***	−0.267 ***	−0.169 ***	−0.216 ***	−0.254 ***
Mental health (MCS12)	−0.321 ***	0.121 **	−0.192 ***	−0.231 ***	−0.204 ***	−0.235 ***	−0.367 ***	−0.218 ***	−0.277 ***

*** *p* < 0.001, ** *p* < 0.01, * *p* < 0.05, FCV1: most afraid, FCV2: uncomfortable, FCV3: clammy hands, FCV4: afraid of dying, FCV5: nervous/anxious, FCV6: can’t sleep, FCV7: heart palpitations.

**Table 4:** Fear for COVID-19 and physical/mental health by demographic and health-related characteristics.

Characteristic		FCV-19s	PCS	MCS	Characteristic		FCV-19s	PCS	MCS
Gender					Comorbidities				
Male		16.65	51.60	49.02	Yes		19.71	45.44	44.43
Female		19.24	49.45	45.41	No		17.98	51.38	47.22
	*p-sig.*	<0.001	0.002	<0.001		*p-sig.*	0.004	<0.001	0.032
Age group					Hospitalized (comorbidity)				
18–24		16.98	53.65	43.70	Yes		23.00	37.17	40.01
25–34		18.22	51.61	46.34	No		18.32	50.32	46.60
35–44		17.58	51.75	45.96		*p-sig.*	0.054	<0.001	0.200
45–54		18.78	49.91	46.86	Contacts with comorbidity				
55–64		18.75	47.89	48.36	Yes		19.27	49.62	45.32
65+		19.77	43.26	47.93	No		17.34	50.86	47.98
	*p-sig.*	0.127	<0.001	0.142		*p-sig.*	<0.001	0.131	0.001
Family status					Contacts with COVID-19				
Single		16.92	51.70	45.95	Yes		18.28	50.24	46.64
Married/Partner		18.78	50.16	47.14	No		18.55	49.75	46.55
Divorced/Widowed		18.47	46.18	44.67		*p-sig.*	0.455	0.510	0.823
	*p-sig.*	0.008	<0.001	0.173	Caught COVID-19				
Education					Yes		17.30	49.70	46.29
≤9 years		20.48	46.21	46.55	No		18.61	50.34	46.76
10–12 years		18.85	49.63	48.13		*p-sig.*	0.041	0.204	0.967
≥ 12 years		17.72	51.11	45.91	Hospitalized for COVID-19				
	*p-sig.*	0.003	<0.001	0.080	Yes		18.44	48.33	47.34
Work status					No		18.31	50.27	46.61
Employed		17.99	50.88	47.06		*p-sig.*	0.780	0.255	0.630
Housekeeping/Retired		21.01	44.93	45.45	Vaccinated for COVID-19				
Unemployed		17.84	51.32	45.13	Yes		18.64	49.95	46.81
	*p-sig.*	<0.001	<0.001	0.262	No		17.09	51.24	46.14
Health change (past year)						*p-sig.*	0.006	0.101	0.776
Much better		15.94	52.32	51.29	Updated about COVID-19				
Somewhat better		17.02	51.48	48.97	Regularly (e.g., daily)		19.65	49.14	46.75
Roughly the same		18.38	50.60	47.00	Often (e.g., 2–3 per week)		17.33	51.03	46.96
Somewhat worse		21.44	43.49	37.46	Rarely (e.g., once a week)		17.59	50.84	44.63
Much worse		20.20	47.62	40.09	Never (only by chance)		15.83	52.12	46.91
	*p-sig.*	<0.001	<0.001	<0.001		*p-sig.*	<0.001	0.001	0.574

Note: Significance is determined by Mann-Whitney and Kruskal Wallis tests.

**Table 5:** PCS and MCS models with Gamma regression.

Dependent Variable	Explanatory Variables	Estimates		
		Beta (std. err.)	t-Value	*p*-Value
PCS	Constant	4.207 (0.034)	107.38	<0.05
	FCV-19s	−0.008 (0.001)	−5.481	<0.001
	Age (years)	−0.003 (0.001)	−4.638	<0.001
	Hospitalized for comorbidity	−0.240 (0.056)	−4.262	<0.001
	Gender (female)	−0.200 (0.013)	−1.390	>0.05
	Comorbidity	−0.035 (0.988)	−1.701	0.089
	Family status			
	Married/Partner	0.039 (0.018)	2.138	<0.05
	Divorced/Windowed	−0.031 (0.291)	−1.075	>0.05
	Past year health change (decreasing)			
	Somewhat better	0.010 (0.034)	0.297	>0.05
	Almost same	−0.017 (0.023)	−0.723	>0.05
	Somewhat worse	−0.115 (0.032)	−3.645	<0.001
	Much worse	−0.060 (0.067)	−0.889	>0.05
MCS	Constant	4.043	72.53	<0.001
	FCV-19s	−0.011 (0.002)	−6.359	<0.001
	Age (years)	0.025 (0.001)	2.553	<0.02
	Hospitalized for comorbidity	−0.047 (0.080)	−0.588	>0.05
	Gender (female)	−0.034 (0.020)	−1.712	<0.10
	Comorbidity	−0.032 (0.029)	−1.089	>0.05
	Family status			
	Married/Partner	0.004 (0.026)	0.164	>0.10
	Divorced/Windowed	−0.075 (0.041)	−1.805	<0.10
	Past year health change (decreasing)			
	Somewhat better	−0.015 (0.048)	−0.322	0.10
	Almost same	−0.070 (0.033)	−2.142	<0.05
	Somewhat worse	−0.266 (0.045)	−5.914	<0.001
	Much worse	−0.172 (0.096)	−1.900	<0.1

## Data Availability

Not applicable.
